# Post-Translational Modification of WRKY Transcription Factors

**DOI:** 10.3390/plants13152040

**Published:** 2024-07-25

**Authors:** Xiangui Zhou, Zaojuan Lei, Pengtian An

**Affiliations:** 1 State Key Laboratory of Protein and Plant Gene Research, School of Advanced Agricultural Sciences and School of Life Sciences, Peking-Tsinghua Center for Life Sciences, Peking University, Beijing 100871, China; 2 Huanghua Port Business Department, Technical Center of Shijiazhuang Customs District, Cangzhou 061113, China; leizaojuan0422@163.com (Z.L.); anpengtian@163.com (P.A.)

**Keywords:** post-translational modification, phosphorylation, ubiquitination, WRKY TFs

## Abstract

Post-translational modifications (PTMs) of proteins are involved in numerous biological processes, including signal transduction, cell cycle regulation, growth and development, and stress responses. WRKY transcription factors (TFs) play significant roles in plant growth, development, and responses to both biotic and abiotic stresses, making them one of the largest and most vital TF families in plants. Recent studies have increasingly highlighted the importance of PTMs of WRKY TFs in various life processes. This review focuses on the recent advancements in understanding the phosphorylation and ubiquitination of WRKY TFs, particularly their roles in resistance to biotic and abiotic stresses and in plant growth and development. Future research directions and prospects in this field are also discussed.

## 1. Introduction

As sessile organisms, plants cannot move and are highly susceptible to external biotic and abiotic stresses. Biotic stresses include attacks by fungi, bacteria, viruses, nematodes, and insects, while abiotic stresses encompass salt, drought, cold, heat, UV-B radiation, and heavy metal exposure. These stresses significantly impact plant growth and development, ultimately leading to reduced crop yields [1]. Over long periods of interaction with their environment, plants have evolved complex and precise systems to adapt to changing conditions. The ability of plants to perceive and respond to stimuli is crucial for their survival. Stimulus perception involves sensors or receptors on the cell surface that detect various stresses and transmit these signals through multiple signaling pathways [2]. A key signaling module downstream of receptor-like protein kinases (RLKs) is the mitogen-activated protein kinase (MAPK) cascade, which acts as a molecular switch in sensing upstream signals and responding to environmental stress. The MAPK cascade phosphorylates various downstream targets, including TFs, structural proteins, protein kinases, and other enzymes [3]. TFs are essential regulatory components in signal transduction. By binding to cis-acting elements in target gene promoters, TFs can enhance or inhibit the transcription efficiency of target genes, thereby enabling adaptive regulation in response to environmental stress. Plant TF families include MYB, bZIP, ERF, NAC, and WRKY, among others. The WRKY TF family is one of the most important and extensively studied families in the plant stress response [4].

The WRKY TF family is large, with numerous members identified across various species due to advances in genomics research. For instance, 102 WRKY TFs have been identified in *Oryza sativa* [5], 72 in *Arabidopsis thaliana* [6], 140 in *Zea mays* [7], and 278 in *Brassica napus* [8]. WRKY TFs play crucial roles in plant immunity [9,10,11,12] and are important regulators of plant growth and development [13,14,15,16,17,18,19]. Additionally, WRKY TFs are involved in responses to abiotic stresses [20,21,22,23]. High-density *Arabidopsis* protein microarrays have been used to identify downstream targets of MAPKs, revealing that TFs such as TGA, MYB, bZIP, and WRKY are potential MAPK substrates [24]. Other protein kinase families, including SnRK2 and CDPK, have also been shown to phosphorylate WRKY TFs [25,26,27,28,29,30]. Furthermore, WRKY TFs can be regulated by ubiquitination [31,32,33,34,35] and persulfidation [36,37]. In this review, we summarize recent findings on the phosphorylation and ubiquitination of WRKY TFs by various protein kinases and their roles in regulating responses to biotic and abiotic stresses, as well as plant growth and development.

## 2. The Function of WRKY’s Phosphorylation in Plants

### 2.1. The Phosphorylation of WRKY Proteins under Biotic Stress

Mitogen-activated protein kinase (MAPK) cascades function downstream of receptors/sensors, transducing extracellular stimuli into cellular responses [38]. The core components of a MAPK cascade include a MAP kinase kinase kinase (MAPKKK or MEKK), a MAP kinase kinase (MAPKK, MKK, or MEK), and a MAP kinase [39]. In *Nicotiana benthamiana*, NbWRKY8 is phosphorylated at clustered proline-directed serines (SP cluster) by SIPK and WIPK (AtMPK3 and AtMPK6 orthologs, respectively). NbWRKY8 interacts with these MAPKs via a MAPK-docking (D) domain, and this interaction is required for effective phosphorylation of NbWRKY8 in plants [9] (Table 1). Moreover, the phospho-mimicking mutant of NbWRKY8 activates the downstream gene *HMGR2*. Group I WRKYs, including AtWRKY25 and NtWRKY1, are also in vitro substrates of MAPKs [40,41].

AtWRKY33, the closest *Arabidopsis* WRKY to NbWRKY8, has been identified as a classic substrate of MPK3/MPK6 [10]. MPK3/MPK6 phosphorylate multiple residues of AtWRKY33 in response to *Botrytis cinerea* infections. Phosphorylation of AtWRKY33 promotes the expression of *PAD3* and is essential for the full induction of camalexin biosynthesis [30]. Recently, WRKY33 was reported to be cooperatively phosphorylated by protein kinases CPK5/CPK6 and MPK3/MPK6 to activate camalexin biosynthesis in *Arabidopsis*, providing protection against *B. cinerea* infection [25], indicating that WRKY TFs function as convergent substrates of differential protein kinases. Additionally, CPK4/5/6/11 phosphorylate AtWRKY8, AtWRKY28, and AtWRKY48, enhancing their binding activity to W-box elements, thereby reprograming the transcription of immune genes [26].

Other substrates of MPK3/6 include WRKY18 and WRKY46. Recent work has revealed that MPK3/MPK6 interact with and phosphorylate WRKY18, which modulates *PP2C5* expression. The MPK3/MPK6-WRKY18-PP2C5 module significantly reduces *Pseudomonas syringae* infection in *Arabidopsis* [42]. Phosphorylation by MPK3/MPK6 has been experimentally demonstrated for 36 WRKY factors [43]. WRKY46 has been identified as an MPK3/6 phospho-target mediating plant defense responses [43]. Such a large number of WRKY TFs can be phosphorylated by MPK3/6, indicating that WRKY TFs are widely involved in MAPK-mediated signaling pathways.

In rice, OsBWMK1 phosphorylates OsWRKY33, which binds to the W-box element in several PR gene promoters [44]. OsWRKY30 is a downstream target of the OsMKK3-OsMPK7 module and enhances resistance to the bacterial pathogen *Xanthomonas oryzae* pv. *oryzae* (*Xoo*) [45]. Two MAPKs, OsMPK4 and OsMPK6, phosphorylate WRKY45 proteins in vitro [46]. Upon activation by salicylic acid (SA), OsWRKY45 promotes the accumulation of its own mRNA and that of the downstream defense-related genes *OsWRKY62*, *OsNAC4*, and *OsHSF1*, enhancing rice disease resistance [47].

OsMKK4-OsMPK3/6, a rice fungal MAMP-responsive MAPK cascade [48], phosphorylates the multiple clustered serine-proline residues of OsWRKY53 in vitro [49]. Overexpression of the phospho-mimic form of OsWRKY53 can activate numerous defense-related gene expressions and enhance rice blast resistance [49].

WRKY also plays an essential role in plant resistance to insect infestation. Rice recognizes signals derived from chewing herbivores and activates OsMPK3 and OsMPK6, which then phosphorylate OsWRKY70. Phosphorylation of OsWRKY70 increases its transactivation activity but not its DNA-binding activity. The OsMPK3/6-OsWRKY70 module elicits the jasmonic acid (JA) signaling pathway to activate defense responses and decreases gibberellin (GA) production to inhibit plant growth [50]. This strategy of prioritizing defense over growth has been reported elsewhere. OsWRKY31 plays a positive role in defending against *Magnaporthe oryzae* rice and affects lateral root development [51]. Phosphorylation and ubiquitination of OsWRKY31 produce an OsMKK10-2-mediated defense response in rice triggered by *Magnaporthe oryzae* [12]. Additionally, the OsMKK10-2-OsMPK3/6-OsWRKY31 module participates in camalexin biosynthesis to regulate defense and growth [12].

SnRK2s have been implicated in plant growth and survival under osmotic stress conditions [52]. The ABA-inducible SnRK2-type kinase SAPK10 phosphorylates WRKY72 at Thr129. SAPK10-mediated phosphorylation impairs the DNA-binding ability of WRKY72 and releases its suppression on *AOS1* and JA biosynthesis [27]. Plant SUCROSE-NONFERMENTING1 (SNF1)-related kinase 1 (SnRK1) plays a central role in maintaining energy homeostasis for growth and survival [53]. In barley, SnRK1 positively regulates disease resistance by interacting with and phosphorylating WRKY3, a repressor of barley disease resistance to *Blumeria graminis* f. sp. *hordei* (*Bgh*) fungus, at Ser83 and Ser112. Phosphorylation of WRKY3 at these sites destabilizes the protein [54]. Recent work has shown that *Bgh* infection induces the expression of multiple MPK genes, including HvMPK4, and leads to the activation of barley MAPKs by phosphorylation. HvMPK4 negatively regulates barley resistance to powdery mildew. Further studies have identified a HvMKK1-HvMPK4-HvWRKY1 module in the barley immune system, where HvMPK4 enhances the DNA-binding ability and transcriptional inhibition activity of HvWRKY1 by phosphorylating three major amino acid sites: Ser122, Thr284, and Ser347, thereby inhibiting barley resistance to powdery fungi [55].

In other crops, including oilseed rape (*Brassica napus*), apple (*Malus pumila*), sweet potato (*Ipomoea batatas*), pepper (*Capsicum annuum*), and chickpea (*Cicer arietinum*), phosphorylation of WRKY TFs by protein kinases has been reported. The BnaMKK5-BnaA06.MPK3/BnaC03.MPK3 module phosphorylates the substrate BnWRKY33, enhancing its transcriptional activity and positively regulating resistance to *Sclerotinia sclerotiorum* in *Brassica napus* [56]. In apples, the MdMEK4-MdMPK3 module interacts with and phosphorylates MdWRKY17. The MdWRKY17 phospho-mimicking mutant enhances DNA-binding activity to MdDMR6, a salicylic acid (SA) degradation gene, resulting in decreased resistance to *Colletotrichum fructicola* [57]. The MAP kinase MdMKK2 phosphorylates MdWRKY71 at its Thr99 and Thr102 residues. Phosphorylation of MdWRKY71 enhances its transcriptional inhibition of MdCERK1, attenuating the inhibition effect of CERK1 on JA synthesis [58]. The sweet potato WRKY TF IbSPF1 specifically interacts with IbMPK3 and IbMPK6, which phosphorylate Ser75 and Ser110 residues of IbSPF1, increasing its binding affinity to the W-box element in target gene promoters. The phospho-mimicking mutant of IbSPF1 shows enhanced resistance to *Pseudomonas syringae* pv. *tabac* [59]. The CaCDPK29-CaWRKY27b module in pepper plants enhances CaWRKY40-mediated defense responses to *Ralstonia solanacearum* infection and high-temperature and high-humidity stress [28]. In chickpeas, the CC-NLR protein forms homocomplexes and interacts with WRKY64, phosphorylating WRKY64 to protect it from ubiquitination and proteasome-mediated degradation. WRKY64 is phosphorylated to enhance its stability and binds to the EDS1 promoter to induce its transcription [60]. The interaction between CaMPK9 and CaWRKY40 depends on two canonical serine residues. Phosphorylation of CaWRKY40 by CaMPK9 is necessary for CaWRKY40 to resist Foc1 infection, and application of the 26S proteasomal inhibitor MG132 restores resistance in the mutated WRKY40 Ser. 224/225 to AA overexpressing chickpea, indicating that such phosphorylation can protect CaWRKY40 from ubiquitination degradation [61].

**Table 1:** The function of WRKY’s phosphorylation in plant biotic stress.

No.	WRKY TFs	Species	Upstream Kinase	Phosphorylation Sites	Target Gene	Pathway	Refs
1	NbWRKY8	*Nicotiana benthamiana*	NbSIPK, NbNTF4, and NbWIPK	Ser-62, Ser-67, Ser-79, Ser-86, and Ser-98	*NbNADP-ME* and *NbHMGR2*	Defense response	[9]
2	AtWRKY25	*Arabidopsis thaliana*	AtMPK4/6	-		Defense response	[40]
3	NbWRKY1	*Nicotiana benthamiana*	NbSIPK			Defense response	[41]
4	AtWRKY33	*Arabidopsis thaliana*	AtMPK3/4/6	Ser-54, Ser-59, Ser-65, Ser-72, and Ser-85	*AtCYP71A13* and *AtPAD3*	Defense response	[10]
5	AtWRKY33	*Arabidopsis thaliana*	AtCPK5/6		*AtPAD3*	Camalexin biosynthesis	[25]
6	AtWRKY18/28 /48	*Arabidopsis thaliana*	AtCPK4/5/6/11		*AtWRKY46*	Defense response	[26]
7	AtWRKY18	*Arabidopsis thaliana*	AtMKK4-AtMPK3/MPK6		*AtAP2C1* and *AtPP2C5*	Defense response	[42]
8	AtWRKY46	*Arabidopsis thaliana*	AtMPK3	Ser168 and Ser250	*AtNHL10*	Defense response	[43]
9	OsWRKY33	*Oryza sativa*	OsBWMK1		*PR1*	Defense response	[44]
10	OsWRKY30	*Oryza sativa*	OsMKK3-OsMPK7		*PR genes*	Disease resistance	[45]
11	OsWRKY45	*Oryza sativa*	OsMPK4 and OsMPK6		*OsWRKY62*, *OsNAC4*, *OsHSF1*, *OsPEN3-like* and *P450*	Disease resistance	[46,47]
12	OsWRKY53	*Oryza sativa*	OsMKK4-OsMPK3/6	Ser43, Ser72, Ser77, Ser89, Ser96, and Ser108	*defense-related genes/momilactone biosynthetic genes*	Disease resistance	[49]
13	OsWRKY70	*Oryza sativa*	OsMPK3/6		*OsHI-LOX*, *OsAOS2*, *OsACS2*, and *OsICS1*	Insect resistance	[50]
14	OsWRKY31	*Oryza sativa*	OsMKK10-2-OsMPK3/4/6	Ser6 and Ser101	*auxin-related genes/defense-related genes*	Disease resistance root growth	[12]
15	OsWRKY72	*Oryza sativa*	OsSAPK10	Thr129	*AOS1*	Bacteria blight resistance	[27]
16	HvWRKY3	*Hordeum vulgare*	HvSnRK1	Ser83 and Ser112		Disease resistance	[54]
17	HvWRKY11	*Hordeum vulgare*	HvMKK1-HvMPK4	Ser122, Thr284, and Ser347	*PR1b*, *PR2*, and *PR5*	Disease resistance	[55]
18	BnWRKY33	*Brassica napus*	BnaA03.MKK5-BnaA06.MPK3 /BnaC03.MPK3	Ser53, Ser58, Ser64, Ser72, and Ser85	*BnPAD3* and *BnCYP71A13*	Disease resistance	[56]
19	MdWRKY17	*Malus domestica Borkh*	MdMEK4-MdMPK3	Ser61, Ser66, Thr73, Ser77, and Ser325	*MdDRM6*	Disease resistance	[57]
20	MdWRKY71	*Malus domestica Borkh*	MdMMK2	Thr99 and Thr102	*MdCERK1*	Disease resistance	[58]
21	IbSPF1	*Ipomoea batatas*	IbMPK3/6	Ser75 and Ser110	*NbPR1a*, *NbPR1c*, *NbPR2*, and *NbPR4*	Disease resistance	[59]
22	CaWRKY27b	*Capsicum annuum*	CaCDPK29	Ser137	*CaWRKY40*	Disease resistance	[28]
23	CaWRKY64	*Cicer arietinum*	CC-NB-ARC-LRR protein	Ser193	*CaEDS1*	Disease resistance	[60]
24	CaWRKY40	*Cicer arietinum*	CaMPK9	Ser224 and Ser225	*CaDefensin* and *CaWRKY33*	Disease resistance	[61]

### 2.2. The Phosphorylation of WRKY Proteins under Abiotic Stress

Drought significantly impacts plant growth and development and is one of the most severe abiotic stresses, leading to substantial crop yield reduction worldwide. Accumulating reports suggest that WRKY TFs are associated with drought tolerance. For example, transgenic rice lines overexpressing OsWRKY30 exhibit enhanced drought tolerance. OsWRKY30 interacts with OsMPK3, OsMPK4, OsMPK7, OsMPK14, OsMPK20-4, and OsMPK20-5 and can be phosphorylated by OsMPK3, OsMPK7, and OsMPK14. The transcriptional activation of OsWRKY30 is impaired when SP is replaced by AP, and overexpression of phospho-dead OsWRKY30 does not improve drought tolerance. These results strongly indicate that phosphorylation of OsWRKY30 by MAPKs is crucial for OsWRKY30 to perform its biological function [21] (Table 2). After wounding treatment, OsMKK4 phosphorylates OsMPK1, which then directly interacts with OsWRKY53. OsMPK1 activated by the constitutively active mutant OsMKK4DD phosphorylates OsWRKY53 [62]. OsWRKY53 is an important TF involved in various stress pathways, including salt stress, the defense response, and the wounding response.

The phytohormone abscisic acid (ABA) is a pivotal regulator of abiotic stress responses in plants, triggering major changes in plant physiology [63]. ABA can induce antioxidant defense-related enzymes such as ascorbate peroxidase (APX), superoxide dismutase (SOD), glutathione peroxidase (GPX), and catalase (CAT) [64]. ZmWRKY104 is a positive regulator of ABA-induced antioxidant defense. ZmWRKY104 physically interacts with ZmMPK6 in vitro and in vivo, and phosphorylation of ZmWRKY104 at Thr-59 by ZmMPK6 plays a key role in the ABA-induced antioxidant defense [23]. In rice, SAPK10 interacts with and phosphorylates WRKY87 at Ser23. OsWRKY87 positively regulates drought and salt tolerance by increasing SOD, CAT, POD, and APX antioxidants [29].

Cotton (*Gossypium hirsutum*) is an important crop worldwide. A regulatory module consisting of GhMAP3K15-GhMKK4-GhMPK6-GhWRKY59-GhDREB2 is involved in controlling the cotton drought response. GhMKK4 activated by GhMAP3K15 can phosphorylate GhMPK6 in an in vitro kinase assay. GhMPK6 interacts with and phosphorylates GhWRKY59, and GhWRKY59 regulates the expression of *GhDREB2*, thereby positively regulating cotton drought responses [22].

**Figure 1:**
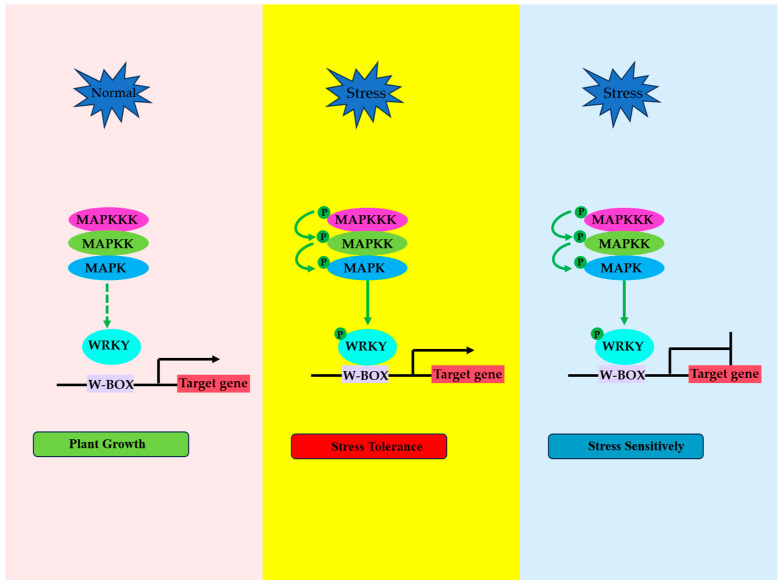
A model of a WRKY TF regulated by protein kinase phosphorylation under stress in plants. Under normal growth conditions, the MAPK cascade signal is not activated, and the binding of WRKY TFs to downstream target gene promoters is little (**left**). Under stress, the activated MAPKKK phosphorylates and activates MAPKK; the activated MAPKK phosphorylates and activates MAPK; and the activated MAPK interacts with and phosphorylates WRKY TFs. The phosphorylated and activated WRKY binds to W-box sites within its target genes and upregulates (**middle**) or downregulates (**right**) the expression of these genes in response to stress.

In summary, the phosphorylation of WRKY TFs by a MAPK cascade or other protein kinases such as SnRK2 and CDPK is in a resting state under normal conditions (Figure 1. left). Under stress, the MAPK cascade phosphorylates the WRKY TF and enhances (Figure 1. middle) or inhibits (Figure 1. right) the transcriptional activation activity and DNA-binding activity of the WRKY TF, following which downstream target genes are upregulated or downregulated to respond to stress (Figure 1). This mechanism is widely applicable, including NbWRKY8 [9], AtWRKY33 [10,25], NbWRKY1 [41], AtWRKY18 [42], AtWRKY46 [43], OsWRKY33 [44], OsWRKY30 [45], OsWRKY45 [46], OsWRKY53 [49], OsWRKY70 [50], OsWRKY31 [12], BnWRKY33 [56], MdWRKY71 [58], IbSPF1 [59], CaWRKY40 [61], OsWRKY30 [21], ZmWRKY104 [23], GhWRKY59 [22], and OsWRKY78 [19].

### 2.3. The Phosphorylation of WRKYs in Plant Growth and Development

Over the past two decades, the function of WRKY TFs in plant defense responses has been extensively studied. Recent findings have revealed the important role of WRKYs in plant growth and development. As sessile organisms, plants require WRKY TFs for balanced growth, development, and stress tolerance. This section discusses the significant role of protein kinases in regulating WRKY TF phosphorylation during plant growth and development.

In *Arabidopsis*, a MAP kinase kinase, MEKK1, can phosphorylate senescence-related WRKY53 in vitro. This phosphorylation increases the DNA-binding activity of WRKY53 in vitro and enhances the transcription of a WRKY53 promoter-driven reporter gene in vivo [65] (Table 3). WRKY34 is phosphorylated by MPK3/6 during the early stages of pollen development, which is crucial for its function in pollen development [14]. WRKY46, WRKY54, and WRKY70 promote BR-mediated gene expression while also inhibiting drought-responsive genes. WRKY54 directly interacts with BES1 to cooperatively regulate the expression of target genes and is phosphorylated and destabilized by BIN2, a negative regulator in the BR pathway [66].

Casein kinase 1 AELs (CK1s) are highly conserved serine/threonine protein kinases in eukaryotes. CK1s regulate blue light signaling by promoting the degradation of CRY2 through phosphorylation and ethylene synthesis by phosphorylating ACS5 [67,68]. Recently, it has been reported that WRKY22 is also a target of CK1. AELs interact with and phosphorylate WRKY22 at Thr57, Thr60, and Ser69 residues to enhance transactivation activity. Increased or suppressed phosphorylation of WRKY22 results in promoted or delayed leaf senescence, respectively. WRKY22 directly binds to the promoter region and stimulates the transcription of the *ACS7* gene, promoting ethylene levels and, consequently, leaf senescence [69].

In rice, OsWRKY53 positively regulates BR signaling. Overexpression of OsWRKY53 leads to enlarged leaf angles and increased grain size, while the *oswrky53* mutant exhibits erect leaves and smaller seeds. OsWRKY53 interacts with and is phosphorylated by the OsMAPKK4-OsMAPK6 cascade. Phosphorylation of OsWRKY53 by MAPK6 is required for its function in BR responses [15]. A complete OsMKKK70-OsMKK4-OsMAPK6-OsWRKY53 signaling pathway regulates grain size and leaf angle in rice. Overexpression of constitutively active forms of OsMKK4, OsMAPK6, and OsWRKY53 can rescue the phenotype of *osmkkk62/70* grain size and leaf angle. These results suggest that OsMKKK70-OsMKK4-OsMAPK6 may function through a common MAPK signaling pathway [16]. OsWRKY53 interacts with OsGSK2, a negative regulator of BR signaling, which reduces its protein stability by phosphorylation. Knockout of OsWRKY53 in OsGSK2-RNAi (Gi) transgenic rice restores the Gi phenotype to wild-type levels. Transcriptome analysis shows that OsWRKY53 and OsGSK2 have opposite regulatory patterns, with WRKY53 restoring the expression of genes regulated by Gi to varying degrees, suggesting that WRKY53 is indispensable for GSK2 function and acts genetically downstream of GSK2 [17].

Panicle exsertion is a crucial agronomic trait in rice, and gibberellin (GA) plays important roles in regulating panicle exsertion. OsWRKY78 affects GA content by directly regulating GA biosynthesis genes and indirectly regulating GA metabolism genes, consequently controlling panicle exsertion. OsWRKY78 interacts with and is phosphorylated by OsMAPK6, and this phosphorylation is indispensable for its biological function. Overexpression of OsWRKY78 (SD), but not OsWRKY78 (SA), can fully rescue the panicle enclosure defect of *oswrky78* and largely complement the decreased height of the *oswrky78* mutant [19].

BnaCPK5 and CPK6 interact with and phosphorylate BnaWSR1. Overexpression of phosphomimic BnaWSR1 in rapeseed protoplasts elicits ROS production and cell death, while its ectopic expression in *Arabidopsis* enhances SA and ROS levels and accelerates leaf senescence [30]. GhWRKY16 is phosphorylated by GhMPK3-1 at residues Thr130 and Ser260. Phosphorylated GhWRKY16 activates four downstream target genes for early fiber development [70]. FvWRKY50 interacts with FvMAPK3 in vitro and in vivo. FvMAPK3 promotes the degradation of the FvWRKY50 protein through the phosphorylation of FvWRKY50 at low temperatures, suggesting that FvWRKY50, as the downstream substrate of FvMAPK3, plays an important role in inhibiting anthocyanin accumulation in strawberry fruit at low temperatures [18].

**Table 3:** The function of WRKY’s phosphorylation in plant growth and development.

No.	WRKY TFs	Species	Upstream Kinase	Phosphorylation Sites	Target Gene	Pathway	Refs
1	AtWRKY53	*Arabidopsis thaliana*	AtMEKK1	-		Senescence	[65]
2	AtWRKY34	*Arabidopsis thaliana*	AtMKK4/5-AtMPK3/6	Ser-87, Ser-91, Ser-98, Ser-108, Ser-274, and Ser-544		Pollen development	[14]
3	AtWRKY54	*Arabidopsis thaliana*	AtBIN2		*AtABI5*, *AtGLY17*, *and AtRD20*	Brassinosteroid-regulated plant growth and drought responses	[66]
4	AtWRKY22	*Arabidopsis thaliana*	AtCK1	Thr57, Thr60, and Ser69	*AtACS7*	Senescence	[69]
5	OsWRKY53	*Oryza sativa*	OsMKKK70-OsMAPKK4-OsMAPK6	Ser43, Ser72, Ser77, Ser89, and Ser96	*OsD2*, *OsDWF4*, *and OsD11*	BR signaling and plant architecture	[15,16]
6	OsWRKY53	*Oryza sativa*	OsGSK2	Thr-236, Thr-252, Ser-322, Ser-323, Ser-373, Thr-379, and Thr-401	*OsD2*, *OsDWF4*, and *OsD11*	Rice architecture and seed size	[17]
7	OsWRKY78	*Oryza sativa*	OsMKK4-OsMAPK6	Ser48, Ser55, Ser67, Ser74, and Ser86	*OsGA20ox-1*, *OsGA20ox-3*, and *OsGA3ox-1*	Panicle exsertion	[19]
8	BnaWSR1	*Brassica napus*	BnaCPK5/6	Thr192 and Thr193	*BnaICS1*, *BnaRboh D*, and *BnaSAG14*	Cell death and leaf senescence	[30]
9	GhWRKY16	*Gossypium hirsutum*	GhMKK2-GhMPK3-1	Thr130 and Ser260	*GhMYB25*, *GhMYB109*, *GhCesA6D-D11*, and *GhHOX3*	Fiber initiation and elongation	[70]
10	FvWRKY50	*Fragaria vesca*	FvMKK4/FvMAPK3		*FvFT2*, *FvCO*, *FvFT3*, *FvSAUR36*, *FvCHI*, and *FvDFR*	Growth and fruit ripening	[18]

## 3. Ubiquitination Modifications and Other Post-Translational Modifications

Ubiquitin modifications are involved in almost all life processes of eukaryotes, and the ubiquitin–proteasome degradation pathway is a crucial mechanism regulating protein expression levels [71]. Studies have shown that ubiquitination can mediate the degradation of TFs in many stress response pathways through the ubiquitin–proteasome degradation pathway, thereby participating in stress responses [31,33,34,72,73]. Additionally, plants accumulate misfolded proteins when subjected to stress, and ubiquitination can also reduce plant damage by degrading these toxic proteins [74].

A previous study showed that PHO1 and WRKY6 play positive and negative roles under low-phosphate (Pi) stress, respectively [75]. After screening 425 homozygous E3 ligase T-DNA insertion mutants, *pru1* exhibited increased sensitivity to low-Pi stress. PRU1 interacts with and polyubiquitinates WRKY6. Under low-phosphate (Pi) stress, PRU1 can ubiquitinate WRKY6 and degrade WRKY6, thereby relieving the transcriptional inhibition effect of WRKY6 on *PHO1*. A genetic analysis confirmed that WRKY6 functioned downstream of PRU1. These results indicated that ubiquitination degradation of WRKY6 by PRU1 was crucial for plant responses to low-Pi stress [31] (Table 4).

WRKY70 is phosphorylated by an unknown protein kinase at Thr22 and Ser34 upon pathogen infection. It binds preferentially to the WT box in the promoter of the defense gene *SARD1* and induces its expression. When the infection ends, non-phosphorylated WRKY70 binds to both W (inhibitory activity site) and WT (active activity site) boxes in the promoter of *SARD1*, thereby shutting down its expression. This is the first report showing that a TF can activate and inhibit the same defense gene in plants. Phosphorylated WRKY70 can be ubiquitinated by the E3 ligase CHYR1 for degradation [32].

WRKY70 is phosphorylated by an unknown protein kinase at Thr22 and Ser34 upon pathogen infection. WRKY70 activates *SARD1* expression by binding to the WT-BOX; phosphorylated WRKY70 was degraded by the 26S proteasome via CHYR1 when normal growth resumed after a pathogen attack. Meanwhile, non-phosphorylated WRKY70 inhibited the expression of *SARD1* by binding to W-BOX (inhibitory activity site) and WT-BOX (active activity site) [32]. The WRKY33/WRKY12-RAP2.2 module plays a key role in the submergence-induced hypoxia response of *Arabidopsis* [76]. The *Arabidopsis* E3 ligase protein SUBMERGENCE RESISTANT1 (SR1) negatively regulates the submergence response by degrading phosphorylated WRKY33 [33]. CONSTITUTIVELY PHOTOMORPHOGENIC 1 (COP1) mediates various cellular and physiological processes in plants by targeting numerous substrates for ubiquitination and degradation [77]. COP1 interacts with and targets WRKY32 for ubiquitination and degradation in darkness [34]. MAX1–MAX4, core components of strigolactone (SL) biosynthesis and signaling, positively regulate freezing tolerance in plants. WRKY41 is targeted for degradation by the F-box protein MAX2. WRKY41 binds to the W-box in CBF promoters and represses CBF expression during cold stress. MAX2 facilitates cold-induced degradation of WRKY41 to release the inhibition of CBFs, thereby enhancing plant freezing tolerance. These results reveal the molecular mechanisms by which SLs regulate plant freezing tolerance via MAX2-dependent degradation of WRKY41, the negative regulator of CBF expression [35].

In rice, OsWRKY7 is a multilayer-regulated disease resistance gene. Under normal conditions, the full-length OsWRKY7 proteins are degraded by the ubiquitin system to reduce adverse effects on plant growth and development. Meanwhile, the short, stable OsWRKY7 protein encoded by diORF, which is generated by alternative translation, provides a constant basal defense. Through gene editing mutation of OsWRKY7 main AUG, the inhibition of downstream diORF translation can be lifted, which leads to the increase in short and stable endogenous expression of OsWRKY7 protein, and then improves the expression of defense genes and ROS levels, and finally strengthens the basal immunity of plants [78].

In wheat, TaSDIR1-4A mediates the ubiquitination degradation of the membrane-bound TF TaWRKY29, leading to its translocation from the membrane to the nucleus, thereby regulating the ABA signaling pathway to improve drought tolerance [79]. TaWRKY74 is a positive regulator of wheat resistance to ‘*Candidatus* Phytoplasma tritici,’ and SWP12 is a potential effector secreted by ‘*Ca.* P. tritici.’ SWP12 targets and degrades TaWRKY74 via the 26S proteasome, suppressing wheat resistance [80].

EIRP1 interacts with VpWRKY11 in vitro and in vivo and mediates its ubiquitination degradation in vivo. VpWRKY11 is a negative regulator of the pathogens *Golovinomyces cichoracearum* and *Pseudomonas syringae* pv *tomato* DC3000 [72].

In woody poplars, PalPUB79 positively regulates drought tolerance in an ABA-dependent manner. PalPUB79 forms a complex with PalWRKY77, mediates its ubiquitination for degradation, and weakens its transcriptional inhibitory effect on PalRD26 [73].

**Figure 2:**
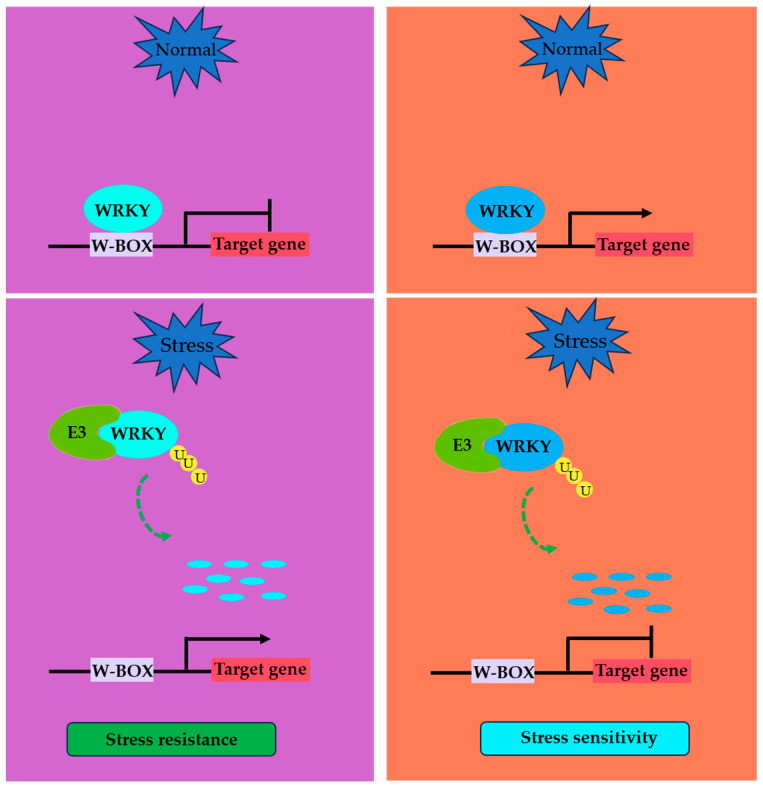
A proposed working model for the function of a E3 ubiquitin ligase as a positive regulator (**left**) and a negative regulator (**right**) targeting a WRKY TF in modulating stress tolerance in plants. Under normal conditions, WRKY TFs acting as negative regulators bind to downstream target gene promoters and repress their expression. When exposed to stress, the expression of E3 ubiquitin ligase as a positive regulatory factor is induced and activated, and the E3 ligase degrades WRKY TFs by ubiquitination, thereby relieving the inhibitory effects of WRKY TFs on downstream target genes and improving resistance to stress (**left**). Under normal conditions, WRKY TFs acting as positive regulators bind to downstream target gene promoters and promote their expression. When exposed to stress, the expression of E3 ubiquitin ligase as a negative regulator is induced and activated, and the E3 ligase degrades WRKY TFs by ubiquitination, thus making WRKY TFs unable to activate downstream target genes and increasing the sensitivity to stress (**right**).

In summary, WRKY TFs can be degraded as negative or positive regulators by different E3 ubiquitin ligases to participate in various stress responses (Figure 2).

Accumulating evidence has shown that approximately 5% of proteins are persulfidated in *Arabidopsis*, a PTM whereby the thiol group at cysteine residues (–SH) is replaced with the persulfidation group (–SSH). A new tag-switch assay revealed that 2015 proteins in *Arabidopsis thaliana* are persulfidated, suggesting that at least 5% of the *A. thaliana* proteome may be persulfidated under non-stress conditions [81]. In tomatoes (*Solanum lycopersicum*), WRKY71 interacts with BRG3, and both proteins are modified by persulfidation. H2S-mediated persulfidation of BRG3 and WRKY71 weakens BRG3′s ubiquitination ability and enhances WRKY71′s binding ability to *CAS1*, thereby delaying tomato fruit ripening [36]. Persulfidation of SlWRKY6 by H2S attenuates its transcriptional activity on target genes *SlSGR1* and *SlSAG12*. However, phosphorylation of SlWRKY6 by SlMAPK4 activates the expression of *SlSGR1* and *SlSAG12*, promoting tomato fruit ripening. Evidence suggests that H2S-mediated persulfidation of SlWRKY6 attenuates SlMAPK4-mediated phosphorylation of SlWRKY6, inhibiting tomato fruit ripening. This study provides a solid theoretical basis for understanding the mechanisms of persulfidation and phosphorylation of SlWRKY6 in regulating tomato fruit ripening [37].

**Table 4:** The function of WRKY’s ubiquitination in plant.

No.	WRKY TFs	Species	E3 Ligase	Pathways	Refs
1	AtWRKY6	*Arabidopsis thaliana*	AtPRU1	Low-Pi stress	[31]
2	AtWRKY70	*Arabidopsis thaliana*	AtCHYR1	Immunity and growth	[32]
3	AtWRKY33	*Arabidopsis thaliana*	AtSR1	Submergence response	[33]
4	AtWRKY32	*Arabidopsis thaliana*	AtCOP1	Photomorphogenesis	[34]
5	AtWRKY41	*Arabidopsis thaliana*	AtMAX2	Freezing tolerance	[35]
6	OsWRKY7	*Oryza sativa*	-	Disease resistance	[78]
7	TaWRKY29	*Triticum aestivum*	TaSDIR1-4A	Drought resistance	[79]
8	TaWRKY74	*Triticum aestivum*	SWP12	Disease resistance	[80]
9	VpWRKY11	*Vitis pseudoreticulata*	EIRP1	Disease resistance	[72]
10	PalWRKY77	*Populus alba*	PalPUB79	Drought tolerance	[73]

## 4. Conclusions and Future Prospects

Following translation, the regulation of protein function primarily depends on PTMs. To date, over 400 different PTMs have been identified in eukaryotes [82]. Among them, the formation and regulatory mechanisms of some PTMs have been investigated in recent years, such as phosphorylation, ubiquitinylation, persulfidation, histone acetylation, methylation, and SUMOylation [83]. Phosphorylation stands out as a major form of PTMs, critically regulating complex signaling networks by phosphorylating a series of substrate proteins. WRKY TFs emerge as pivotal players in plant responses to both biotic and abiotic stresses, as well as growth and development. This review emphasizes the significant role of PTMs, especially phosphorylation and ubiquitinylation, in regulating responses to biotic and abiotic stresses, as well as growth and development, mediated by WRKY TFs, and summarizes the target genes and signaling pathways involved. Despite the abundance of WRKY TFs in plants, most studies are focused on the model plant *Arabidopsis*. In future studies, more attention should be directed towards investigating WRKY genes in crops. Indeed, the utilization of transcriptomics, metabolomics, proteomics, and Chip-seq has facilitated the identification of WRKY-regulated gene networks and metabolic networks, interacting proteins, in vivo target genes, and potential signaling pathways. Among these interacting proteins, particular emphasis should be placed on protein kinases, as existing reports provide ample evidence that phosphorylated, activated forms of WRKY play a crucial role in plant resistance to biotic and abiotic stresses and growth and development. In the future, with the ongoing advancement of CRISPR-Cas9 gene editing technology, targeted mutations of WRKY TF phosphorylation sites can be carried out to develop crop varieties with desirable traits such as high yield, quality, disease resistance, and stress tolerance.

## Figures and Tables

**Table 2:** The function of WRKY’s phosphorylation in plant abiotic stress.

No.	WRKY TFs	Species	Upstream Kinase	Phosphorylation Sites	Target Gene	Pathway	Refs
1	OsWRKY30	*Oryza sativa*	OsMPK3, OsMPK7, and OsMPK14	Ser18, Ser20, Ser120, Ser129, Ser136, Ser148, Ser251, and Ser623	*WSI76*, *WRKY11*, *OsISAP1*, *Oshox7*, *HSP (LOC_Os03g16020)*, and *HSP (LOC_Os09g31486)*	Drought tolerance	[21]
2	OsWRKY53	*Oryza sativa*	OsMKK4-OsMPK1	-		Wounding response	[62]
3	ZmWRKY104	*Zea mays*	ZmMPK6	Thr59		Drought tolerance	[23]
4	OsWRKY87	*Oryza sativa*	OsSAPK10	Ser23	*OsABF1*	Drought and salinity tolerance	[29]
5	GhWRKY59	*Gossypium hirsutum*	GhMAP3K15-GhMKK4-GhMPK6	Ser221	*GhDREB2*	Drought tolerance	[22]
